# Increased grey wolf diurnality in southern Europe under human-restricted conditions

**DOI:** 10.1093/jmammal/gyad003

**Published:** 2023-03-23

**Authors:** Alejandro Martínez-Abraín, Ánxela Llinares, Luis Llaneza, Pilar Santidrián Tomillo, Juan Pita-Romero, Ramón J Valle-García, Victoria Formoso-Freire, Alejandra Perina, Daniel Oro

**Affiliations:** Universidade da Coruña, Facultade de Ciencias, Campus da Zapateira s/n, 15008 A Coruña, Spain; Universidade da Coruña, Facultade de Ciencias, Campus da Zapateira s/n, 15008 A Coruña, Spain; Universidade da Coruña, Facultade de Ciencias, Campus da Zapateira s/n, 15008 A Coruña, Spain; A.RE.NA Asesores en Recursos Naturales, S.L. Perpetuo Socorro 12, Entresuelo 2B, 27003 Lugo, Spain; Animal Demography and Ecology Unit, Institut Mediterrani d’Estudis Avançats (CSIC-UIB), 07190 Esporles, Mallorca, Spain; Rúa María Pita 32, 1ºC, 15570 Narón, A Coruña, Spain; Endesa Generación S.A., Departamento de Medio Ambiente, C/ A Balsa s/n, 15320 As Pontes de García Rodríguez, A Coruña, Spain; Universidade da Coruña, Facultade de Ciencias, Campus da Zapateira s/n, 15008 A Coruña, Spain; AllGenetics and Biology S.L., Cubelos, 21, bajo A2, Perillo, 15172 Oleiros, A Coruña, Spain; Centro de Estudios Avanzados de Blanes CEAB (CSIC), 17300 Blanes, Girona, Spain

**Keywords:** diurnality, Grey Wolf, human landscapes, human, wildlife coexistence, nocturnality

## Abstract

Wolves have been the archetype of wildlife persecution by humans for centuries all over the world, and still are heavily persecuted in some regions. Facultative diurnal/nocturnal wild mammals are known to become more nocturnal when persecuted. Conversely, little is known regarding the possibility of wolves becoming more diurnal if not persecuted. We took advantage of a 9-year natural experiment of restricted human access to a restored coal mine debris dump to study the daily activity patterns of wolves under conditions of infrequent human presence. Results were compared with a paired control site with frequent human use. Circadian wolf activity was monitored using camera traps (3 years in human-restricted site; 2 years in control). Additionally, data from two GPS–GSM-collared wolves monitored in a second control site were also analyzed. In our control sites, wolves were nearly inactive during daylight hours. In contrast, in the human-restricted site wolves extended their activity toward noon, with a daily activity peak between 10:00 and 12:00, and showed some activity throughout the entire circadian 2-h interval cycle considered. Wolves clearly had higher diurnality in the human-restricted area with 78% greater incidence of capture with remote cameras during the day than in the control site. We suggest that the shift toward increased diurnality was related to the loss of fear of humans. Evidence in support of this hypothesis comes from flight initiation distance (FID) data. Wolves showed relatively short FIDs when faced with a human observer (range 70–183 m) in broad daylight at the human-restricted site, but were so afraid of humans in the control site that we were unable to conduct FID trials there. Based on these results, we suggest that wolves may increase their diurnality in those European countries with currently increasing movement of human populations from rural to urban areas and that do not conduct lethal control of wolves. This would represent a historical landmark for a species that has been persecuted for many centuries. However, such behavioral shifts could bring new human–wolf conflicts that would require new policies.

Nocturnality in medium- and large-bodied mammals can increase substantially with human disturbance. Gaynor et al. (2018) reviewed daily activity across gradients of human disturbance and found that nocturnality increased in medium- and large-bodied mammals around the world by 36% from conditions of low disturbance to conditions of high disturbance. The latter situation is typical of countries with subsistence or agricultural economies, where predators and large herbivores are seen as enemies or valued resources, respectively. Nocturnality provides for temporal partitioning of a given space that prevents human–wildlife conflict within the context of normal human activity (Dennehy et al. 2021). However, the converse process is also true, with nocturnal mammals that have been persecuted over centuries becoming more diurnal once human persecution is halted, a process that is currently ongoing in most of Europe and North America (Sterba 2012; Martínez-Abraín and Oro 2018; Silliman et al. 2018; Martínez-Abraín et al. 2019a; Cimatti et al. 2021). For example, Eurasian otters (*Lutra lutra*), seldom seen foraging during the day in the recent past in European countries, can now be studied by direct diurnal observation of individuals in the wild at relatively short distances in human-made reservoirs (Llinares et al. 2019). This has happened to some extent as an ecological consequence of the human depopulation of rural areas, together with changing attitudes toward wildlife of modern urban people (Martínez-Abraín et al. 2019a, 2020, 2021) no longer perceiving wildlife to be competitors or enemies. However, in portions of Europe and North America, large predators that are still being persecuted manage to persist in human-dominated landscapes mainly by being shy, nocturnal, and concealed (Zedrosser et al. 2011; Sterba 2012). This is true for European grey wolves (*Canis lupus*), wherein centuries of intense legal and illegal persecution persist into modern times (Theuerkauf 2009; Llaneza et al. 2012; Wam et al. 2012).

France represents a paradigmatic example of wolf persecution, where at the beginning of the 9th century a state-paid specialized corps of wolf hunters was created. It took 1,000 years to extirpate wolves from France using traps, poison, and firearms (Flannery 2018). In Spain, the first known state regulations for the direct persecution of wolves began during the 16th century (1542). From 1953 to 1970, a Spanish state agency was in charge of extirpating wolves and other vertebrate species considered detrimental for game species. Some 2,000 wolves were culled across one-fourth of the Spanish territory in the nine years following its creation (Martínez-Abraín et al. 2020), a figure that is comparatively small compared with the estimated 15,000 wolves killed in Spain between 1855 and 1859 (Rico and Torrente 2000). Human presence in rural areas can be expected to decrease as trends toward an increase in levels of urbanization continues, and as a consequence of this trend wolf behavioral patterns are likely to shift. A characterization of changing behaviors is relevant to establishing effective conservation policy. For example, increased diurnality could lead to new types of human–wolf conflict, such as generating a false sense of wolf overpopulation, even in the absence of wolf population increases.

We took advantage of a natural (i.e., unplanned) human-restriction experiment occurring in an area of high wolf density in northwest (NW) Spain, allowing us to compare circadian rhythms of wolf activity between the human-restricted site and two control sites with higher levels of human presence, located in areas of intensive human land use. We hypothesized that wolves would increase levels of diurnal activity in the exclusion area and more temporal avoidance of humans in the control sites.

To quantify whether wolf nocturnality/diurnality patterns differed between treatment and control areas, we measured wolf activity patterns using camera traps. To test whether wolves in less human-dominated areas exhibited less fear of humans, we also measured flight initiation distance (FID) in the treatment site as a proxy of the level of fear. Measuring FIDs in the control sites was nonviable because wolves were never seen during the day.

Finally, we used our results to identify European countries where wolf diurnality could potentially increase provided that current trends of movement of human populations from rural to urban settings continue into the near future.

## Materials and Methods

### Study sites

**Fig. 1.**
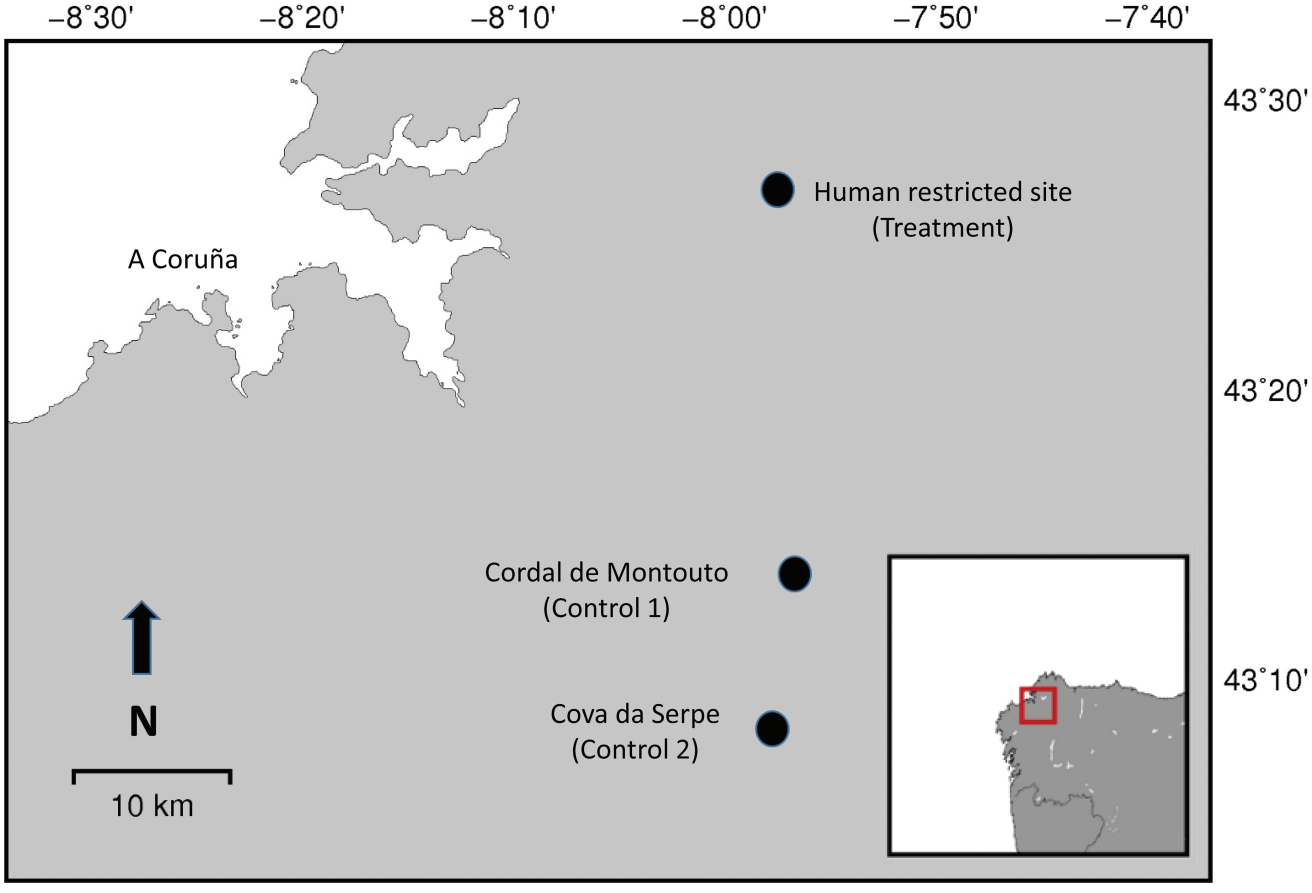
Location of the main study sites in the northwestern region of the Iberian Peninsula.

The human-restricted site (AP) was located close to the town of As Pontes de García Rodríguez, within the province of A Coruña in NW Spain (Fig. 1). The site consists of a large restored and fenced debris dump (1,120 ha; 600 m maximum elevation) from a former coal mine with an adjacent coal-fired thermal plant, and is managed by Endesa Generación S.A. (a major energy power producer in Spain). Restoration operations of the mine started in 1985 and were finished by 2006, 9 years before the onset of the study. The area was ecologically restored, planting both autochthonous and allochthonous vegetation, although large expanses were periodically mowed to prevent forest encroachment in order to maintain grassland habitats and reduce the risk of large forest fires. The resulting landscape is a mosaic of meadows and forested areas with some interspersed lagoons. Although the site is fenced to prevent human access, with a canal defining a portion of the perimeter, large wildlife can access the area through many points in which the fence is permeable while still restricting access for people. Large- and medium-sized mammals now present in the fenced area naturally recolonized the area after restoration and include: Red Deer (*Cervus elaphus*); Roe Deer (*Capreolus capreolus*); Wild Boar (*Sus scrofa*); Red Fox (*Vulpes vulpes*), and Grey Wolf. Horses and cattle are excluded from the site. Wolves use the area as a resting place with opportunistic hunting of young Wild Boar and Red Deer and occasional breeding. No humans can enter the enclosure except for the staff in charge of management or surveillance. Guided visits in a motor vehicle are scheduled from time to time for viewing results of the ecological restoration, but visitors never leave the unpaved road during the visits. AP is monitored by professional security guards on a 24-h basis to prevent human intrusion and has a video-surveillance system. Campaigns to cull wild boars to control their abundance have been conducted by managers every 2 weeks since 2013. Dead wild boars are removed—hence carcasses are not available for wolves or other scavengers. Information on the daily activity patterns of wolves was collected continuously from September 2015 to December 2018 using camera traps (models Ltl Acorn 6210; Browning Spec Ops FHD; Browning Dark Ops Pro XD; Bushnell Agressor HD; Keepguard 760).

Data from the human-restricted site were contrasted with similar information (camera trapping; Moultrie M-880i; Moultrie A-25 Trail) from a control site (Cordal de Montouto, CM) located 25 km away (Fig. 1). This CM control site is a small mountain range (maximum height ca. 700 m) located along the border between the provinces of A Coruña and Lugo. Human population density in the closest main municipality (Guitiriz) was 18.6 inhabitants/km^2^ in 2021. CM mainly consists of pine plantations, meso-hygrophilous scrublands, and cropland devoted to cattle ranching. CM was sampled continuously for approximately two and a half years (July 2017 to December 2019). Importantly, no hunting activity occurs at the control site despite being a terrain with official hunting rights (the Guitiriz TECOR, Lugo province). However, big game (Wild Boar and Roe Deer are common in the area, but Red Deer is rare) is harvested during the hunting season (running October to January) in an adjacent game state belonging to a different province (San Pedro de Cambas TECOR, province of A Coruña). Occasional wolf population controls can be authorized by the regional government in the municipality where the control site is located if wolves kill livestock or domestic dogs. This was not the case during the study period but happened in 2014. Wolves use this site mainly as a resting site, with occasional breeding and hunting, although their diet consists mainly of carrion or livestock from the numerous nearby cattle farms.

We provide complementary information to the camera-trap study consisting of two wolves tracked with GPS–GSM collars (Global Position System–Global System for Mobile communication) as part of a companion study. These wolves were captured and marked at Serra da Cova da Serpe (CS), a small mountain range located 15 km away from our main control site (Fig. 1), with very similar environmental characteristics compared to the main control site (i.e., a low altitude mountain range surrounded by a mosaic of cattle farms and tree and forage crop plantations). As with the other sites, wolves used this site mostly for resting, and only secondarily as hunting grounds and breeding sites.

### Camera-trap settings

We deployed simultaneously a maximum of six cameras (range 1–6; *n* = 15 locations) at AP, and a maximum of six cameras at the control site (range 1–6; *n* = 60 locations) to compare circadian (24 h) wolf activity patterns during a median period of 23 days in both sites (range for 90% of the cases: 14–28 for AP, and 7–88 for CM). Camera sensitivity was set at maximum level, and camera locations were baited only at the beginning of the study.

At AP camera traps were deployed on trees and poles (at a height of approximately 0.5–1.0 m above ground level) along unpaved roads in sites with clear signs of wolf presence (i.e., with footprints, scants, hair, or urine), usually coinciding with crossroads. The area covered was approximately 4.6 km^2^. Minimum separation between camera locations was 227 m. A 30-s video was triggered automatically 0.4 s after detection, with a latent period of 2 s between sequential videos. Only wolf pictures were saved on this site due to logistic problems.

At CM cameras were deployed on trees (at a variable height above the ground ranging from 0.5 to 2 m) at the edges of unpaved roads, where signs of wolf presence were previously detected. Wolf presence signs were known to be common along roads in the study area, and hence wolf detectability is deemed to be high (e.g., Bischof et al. 2014). Cameras were moved to a different location if no wolves were detected within 2 weeks of deployment, if they were detected by people, or if the choice of location was deemed inappropriate in relation to environmental factors (i.e., sunlight, wind, rain, interference by vegetation). A picture was taken automatically after 0.4 s of detection, followed by a 30-s video if the individual remained more than 10 s within the camera range. The latent period between triggers was 2 s. Minimum separation between camera locations was 155 m. The area covered was approximately 600 ha.

To assess the level of diurnality of wolves we recorded the time when one or more wolves were detected by the camera traps. When images of the same wolf or wolves were taken consecutively, we counted only one picture to avoid considering the same individual more than once whenever possible. On some occasions, a detection included more than one individual, but it was recorded just as one detection. Most often we were not able to distinguish one individual from another, and hence all camera records were used. This has the advantage of accounting for the interindividual variability in activity rhythms that may exist. True solar time (rather than corrected clock time) was used in all cases. At CM we saved all pictures and videos of wolves, but also of medium-sized predators, large herbivores, and people. Human visitation is common across all days of the week (i.e., including in all-terrain vehicles, biking, trekking, planting trees, maintaining wind farms, clearing forest vegetation, and horse riding).

### Collared wolves from a second control site

The two collared wolves (one male and one female) from CS were monitored from April to October 2006 and from June to October 2011. Wolves in CS were captured with Belisle leg-hold snares and equipped with GPS–GSM collars from the Swedish enterprise Followit (models Tellus T5H #1367 for the male and Tellus T3H #2396 for the female). Wolves were captured under permits 19/2006 and 86/2011 from the Regional Government of Galicia (i.e., Xunta de Galicia). All fieldwork procedures adhered to national animal welfare regulations. Research followed ASM guidelines (Sikes et al. 2016). GPS collars were scheduled to take a position every 2 h on a daily basis. Information from collared wolves was used to verify whether the information provided by two different monitoring methods (camera trapping at CM and GPS–GSM monitoring at CS) showed similar patterns, which would confirm the reliability of our camera-trap data, because both control sites are close to each other and have quite similar environmental characteristics. We computed the mean of the activity sensor value obtained every 2-h time period.

### Flight initiation distance

We measured FID of wolves and all other medium to large mammals (i.e., Red Deer, Roe Deer, Wild Boar, and Red Fox) observed during the day within the human-restricted site. The study of FIDs consisted of 51 on foot surveys carried out between July 2018 and March 2019. Itineraries were performed between 7:00–11:00 and 19:30–21:30 in summer, and between 8:30–12:00 and 17:30–19:00 in the fall and winter. To get information on FID under conditions of nonhuman restriction we performed 20 on foot surveys adjacent to but outside of the fenced area from June 2020 to August 2020, which is composed mainly of shrublands (*Ulex* and *Erica* spp.) and meadows, with some pine forest areas, and feral horses are abundant. FIDs were recorded employing a laser range finder directly approaching each individual detected along a straight line and recording both the distance at which the individual was initially detected (*D*_0_) and the distance at which each individual fled (FID), following Blumstein (2006) and Møller and Ibáñez-Álamo (2012).

### Data analysis

Pearson’s Chi-squared tests with Yate’s correction together with its residuals were used to analyze differences in wolf activity patterns between control and treatment. The number of wolf detections was binned over 4-h periods and then compared within each period across control and treatment. All statistical analyses were performed using the software environment R (https://www.r-project.org/).

## Results

**Fig. 2.**
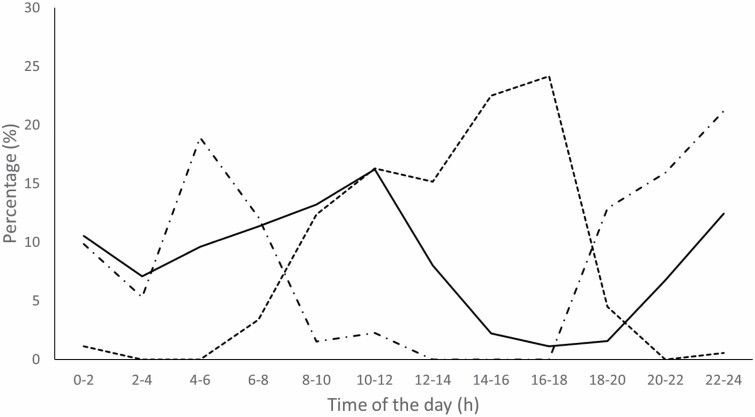
Camera-trap data classified in 2-h intervals used to assess: circadian wolf activity in our main control site (Cordal de Montouto, broken black line with dots; *n* = 132); circadian human activity in the control site (broken black line, *n* = 178); and circadian wolf activity at the human-exclusion site (solid black line, *n* = 636).

**Fig. 3.**
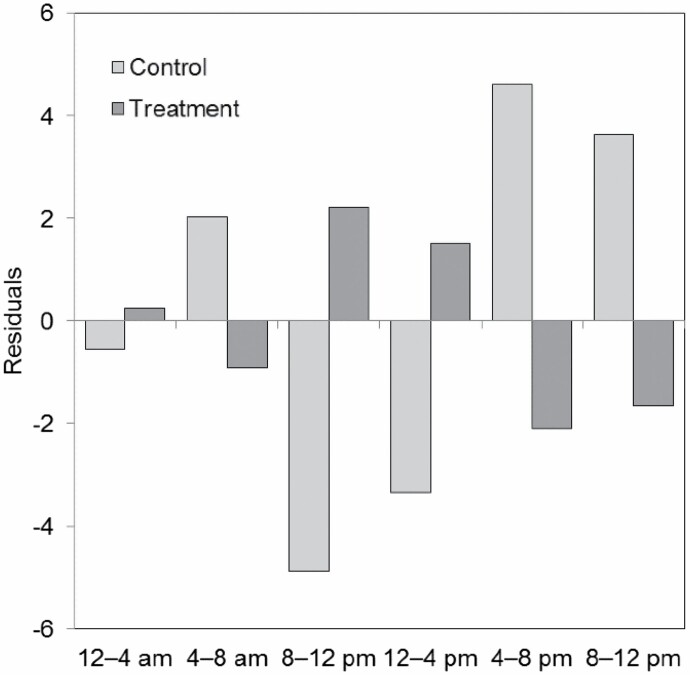
Residuals of a Chi-square test contrasting wolf activity in 4-h periods between the control site (Cordal de Montouto, light gray), and the treatment or human-exclusion site (AP, dark gray). Positive residual values indicate that wolves were more active than expected at each time interval, and negative values indicate that they were less active than expected.

The activity pattern of wolves recorded by camera trapping at CM showed that they were mainly nocturnal (Fig. 2), mostly active from 20:00 to 8:00. Little to no activity was recorded from 8:00 to 18:00, coinciding with the period in which human activity was highest (Fig. 2). However, wolves from AP were relatively inactive between 14:00 and 20:00, although remained active through all 2-h time intervals of the day (Fig. 2). That is, wolves from the human-restricted site were active overnight but continued their activity until 14:00, clearly extending their period of activity into diurnal hours, with a daily activity peak from 10:00 to 12:00. Results of a Chi-square test contrasting activity patterns in periods of 4 h between control (CM) and treatment (AP) showed that wolves at AP were more active than expected from 8:00 to 12:00 and from 12:00 to 16:00 (χ^2^ = 89.05, d.f. = 5, *P* < 0.05; Fig. 3). Overall, the percentage of detections where wolves were active during the day was 20.5% in our main control site versus 36.5% at the human-restricted site (χ^2^ = 11.85, d.f. = 1, *P* < 0.05). Thus, wolf diurnality was 78% higher at the human-restricted site.

Detection rates (wolf detections/day after standardizing the absolute number of wolf detections by the number of days in which cameras were active in both sites) were 0.18 at CM and 0.58 at AP. The minimum number of wolves in AP was 11 versus 5 at CM, judging from the maximum number of wolves detected in a single picture/video in both sites.

**Fig. 4.**
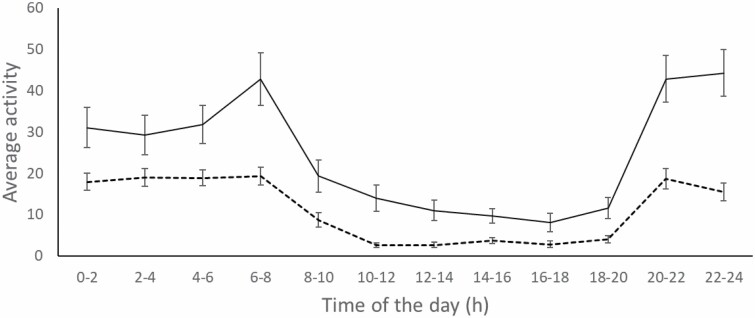
Circadian activity of two adult GPS–GSM-collared wolves (male = dotted black line; female = solid black line) at Serra da Cova da Serpe (second control site). Vertical error bars are standard errors.

Wolf circadian rhythms at CM were highly coincident with the complementary information provided by the two GPS–GSM-collared wolves at CS, suggesting that camera traps were a reliable method to record daily wolf activity at CM (Fig. 4).

Wolves at AP had a mean FID of 107.3 ± 48.90 m (mean ± *SD*, *n* = 4, range = 70–183 m; Table 1). Red Foxes, Red Deer, Roe Deer, and Wild Boar were also observed during the itineraries performed during diurnal hours. Mean wolf FID was shorter than that of Red Deer and Red Foxes, although FID/*D*_0_ ratios were smaller for all species other than grey wolves (Table 1), indicating a shorter waiting time before fleeing when faced with a human approach as an FID/*D*_0_ = 1 means that individuals flew away from the very moment in which they were first detected by the observer. All itineraries performed outside AP provided no sightings of any focal species including wolves.

**Table 1.** Flight initiation distance or FID (arithmetic mean and standard deviation, *SD*) of large mammalian herbivores and carnivores observed inside the human-exclusion site. *D*_0_ is the mean distance at which each individual was initially detected. *N* is the number of observations for each species. An FID/*D*_0_ = 1 indicates that individuals flew away from the very moment in which they were first detected. The smaller the ratio the longer it took them to fly away when faced with an approaching observer.

Species	*n*	FID	*SD*	*D*_0_	FID/*D*_0_
Red deer	103	118.4	68.83	130.1	0.91
Roe deer	30	74.0	43.61	83.1	0.89
Wild boar	132	69.6	64.93	90.4	0.77
Red fox	5	124.0	75.90	145.9	0.85
Grey wolf	4	107.3	48.90	114.1	0.94

## Discussion

Intended large-scale and long-term experiments involving human exclusion/restriction from natural areas are not logistically feasible nor desirable. Situations forcing large-scale human exclusion (such as the Chernobyl power plant accident in Ukraine, the Korean Demilitarized Zone, or the abrupt reduction of human population in Europe caused by the Black Death in the 14th century) are thus rare scenarios across the planet and over human history. However, they provide opportunities, as ‘natural experiments’ resulting in unplanned human exclusion or heavy restriction. The As Pontes coal mine debris dump offered this kind of rare opportunity to investigate the hypothesis that wolves shift activity patterns toward greater diurnality under conditions of human restriction.

According to our camera-trap results, wolves in our CM site were mostly nocturnal, being active mostly outside the hours of major human activity, thus avoiding people through temporal segregation. Monitoring activity patterns with GPS–GSM collars for two wolves supported the robustness of results obtained by camera trapping at CM. Wolf activity rhythms most likely had little causal association with the activity of large wild herbivores present at CM (also mainly nocturnal), because wolves in this area with a number of cattle farms are known to forage mainly on livestock carrion, potentially generating conflicts with cattle owners (Lázaro 2014; Lagos and Bárcena 2015; Llaneza and López-Bao 2015).

While our control wolves were ca. 80% more active at night than during the day, Dalmatian wolves (Croatia) avoided humans by being 51% more active during the night than during the day (Kusak et al. 2005). Wolves in a wildland–agriculture matrix in northwestern Minnesota (Midwest, United States) were also more active at night, when human activity was low (Chavez and Gese 2006); but Mech (1992) recorded higher levels of daytime activity (compared to nightime) of wolves in the Superior National Forest in northeastern Minnesota. In a mainly forested area of the Polish Carpathians (SE Poland; 44 human inhabitants/km^2^) wolves were found to be nocturnal most of the year, but becoming more diurnal during the reproductive season (summer).

On the contrary, at our AP site wolves expanded their activity pattern toward noon, likely being driven by human restriction. This is consistent with the fact that wolves are known to be active throughout the day in areas with low human density, such as Alaska in the Arctic (Mech 1997; Mech et al. 1998) or Białowieza National Park in the temperate forests of Poland (Theuerkauf et al. 2003). However, they are mostly nocturnal when present in human-dominated landscapes (Ciucci et al. 1997; Zedrosser et al. 2011; Wam et al. 2012). Importantly, since cameras were placed in the area used as a daily refuge (because wolves forage outside the refuge areas in both sites), most nocturnal activity records correspond to wolves leaving and entering the refuge areas on their way to foraging grounds.

Large mammalian herbivores were active during daylight hours at AP (Table 1). However, wolves likely did not increase their diurnality because of that. Prey diurnality is a necessary but not sufficient condition to explain wolf shifts toward diurnality. Wolves also need to lose the fear of being detected by humans to be active during the day. At AP, wolves had shorter FIDs than red foxes and red deer, although the wolf FID/*D*_0_ ratio was higher than those of foxes and deer, indicating that wolves took less time to run away since first detected by the observer. The mean wolf FID distance (107 m) was similar to that estimated in low human-population density Canadian ecosystems (mean FID = 106 m; Karlsson et al. 2007), and was similar to that of active Scandinavian brown bears from low human-density landscapes (115 m; Moen et al. 2012). Additionally, wolves were observed four times in broad light during the 51 surveys performed over a period of 8 months, including one observation at a distance of only 70 m from the observer, which certainly reflects a low degree of fear of humans. Although four observations may seem like small sample size, this type of encounter never occurred at CM, where no wolves were observed during the two years of the study. Likewise, no wolves or any other large mammals were observed in the 20 surveys performed outside AP, indicating a high individual behavioral plasticity of wolves as they can change activity patterns depending on local conditions. However, we have recorded the whole pack leaving AP at night and returning at dawn, most likely to hunt foals and calves of the large number of feral horses and cattle nearby (but not within AP), and to rest safely within AP during the day. These are the most common herbivore prey in wolf populations located in highly humanized landscapes in NW Iberia, in which large wild herbivores are either not present or not abundant (Llaneza and López-Vao 2015). Wolves do prey upon the abundant Red Deer and Wild Boar juveniles inside AP, but the most cost-effective prey (i.e., foals and calves) are available outside of this site. Hence, shifts in prey time schedules within AP cannot be the main explanation for shifts in wolf activity patterns. Moreover, wolves inside this site have been observed in broad light following operating mowers to capture unprotected small prey left exposed by the machines, which strongly supports the hypothesis of loss of fear to unthreatening humans.

The most relevant factor driving nocturnality/diurnality was seemingly the regular presence/absence of humans rather than the presence/absence of hunting activity because periodic culling of Red Deer and Wild Boar are scheduled at AP. Cases of carnivores losing fear despite the presence of hunting activity have been reported previously, associated with the availability of food from anthropogenic origins (Swenson 1999). Wolves at CM most likely were afraid of humans because frequent human presence was likely perceived as a risk, especially because these wolves inhabit a human-dominated landscape, and their food is often obtained close to livestock farms.

### From the local to the general pattern

Wolves became more diurnal in the site where a 9-year human restriction was applied, although we cannot tell when the shift happened or if it was gradual or punctuated. However, the rate of change toward diurnality could have been relatively fast, judging by the cases of wolves reported by the mass and social media inside villages and in broad light in NW Spain during the total or partial periods of human lockdown (ca. 2 months) in response to the COVID-19 pandemic. Indeed, a general pattern of increased wildlife diurnality was observed during the COVID-19 lockdown (e.g., Manenti et al. 2020). A rapid change could happen via cultural habituation, copying, and learning, even in shy-selected wolf populations, such as those subjected to intense official human persecution for many centuries in Europe (Martínez-Abraín et al. 2019; Zedrosser et al. 2011).

Over the last six decades, a large-scale nonplanned experiment of human depopulation of rural areas and associated natural rewilding has been taking place in many European countries (Chapron et al. 2014; Martínez-Abraín et al. 2020, 2021; Navarro and Pereira 2012). We suggest that the strong reduction of human density in other rural areas could have similar results to those found in our study, but at a much larger geographical scale. Rural depopulation may result in increased diurnality of some formerly persecuted large- and medium-sized predators, which may no longer strive to avoid human presence through temporal segregation (Carter and Linnell 2016; Chapron and López-Bao 2016; Haswell et al. 2020). Wolves in countries such as Spain, Portugal, Italy, Germany, Poland, Belgium, Denmark, and the Czech Republic could become increasingly diurnal in the near future because these countries show strong decreasing trends in rural population densities, and additionally have (or may have soon) absence of large-scale hunting or lethal control of the species (Table 2; data sources: European Commission; International Wolf Center; web pages for individual countries; The World Bank). The population of wolves in the latter set of eight countries represents 40–45% of the total wolf population currently estimated in the European Union for the period 2013–2018 (https://nature-art17.eionet.europa.eu/article17/species/report/). Hence almost half the European wolf population could become less concealed, increasing the chances of being observed during touristic or recreational activities (Martínez-Abraín et al. 2008). The downside is that if an increase in diurnality eventually takes place, a new type of human–wolf conflict might arise, and new policies (see, e.g., Martínez-Abraín et al. 2019b) might be necessary to allow human–wolf coexistence (Rosenzweig 2003; Carter and Linnell 2016; Chapron and López-Bao 2016). Wolves that inhabit highly human-dominated landscapes exploiting garbage, carrion, or livestock are very close to people (Llaneza et al. 2012, 2016), but they often pass unnoticed by being active at night. A similar use of these resources but in broad light (even without an increase in population size) could alarm people, giving a false perception of wolf overabundance. Specific policies for properly managing garbage, carrion around farms, or even pet food would have to be developed and implemented, as food availability is the main attraction of large carnivores to urban areas (e.g., Braczhowski et al. 2018; Blanco et al. 2021).

**Table 2.** Expected behavior (nocturnality/increased diurnality) of wolves in countries of the European Union based on percentage of human population inhabiting rural areas and its trend, and on the degree of direct human persecution. Increased diurnality (in bold) is to be expected with shrinking rural populations and the absence of hunting or culling. (—) indicates information that was not available. Arrows ↑ and ↓ show the trend (increasing and decreasing, respectively) in the percentage of the rural population over time in each country (data from 2019). The same arrows are used to express the expected trend in wolf behavior in some countries.

Country	Hunting/culling	% Rural population	Expectancy
**Spain**	No hunting allowed (recent Order TED/980/2021, September 20). Official cullings can be authorized.	19%↓	**↑diurnality**
**Portugal**	No	34%↓	**↑diurnality**
France	Culling	19%↓	Nocturnality
**Italy**	No	29%↓	**↑diurnality**
**Germany**	No	23%↓	↑**diurnality**
Switzerland	Culling	26%↓	Nocturnality
Netherlands	—	8%↓	—
**Belgium**	No	2%↓	**↑diurnality**
Norway	Culling	17%↓	Nocturnality
Sweden	Culling	12%↓	Nocturnality
**Denmark**	No	12%↓	**↑diurnality**
Finland	Hunting	15%↓	Nocturnality
Greece	North of 39°N: hunting; South of 39°N: culling	21%↓	Nocturnality
Esthonia	Hunting	31%↓	Nocturnality
Latvia	Hunting	32%↓	Nocturnality
Lithuania	Hunting	32%↓	Nocturnality
Slovenia	Culling	45%↓	Nocturnality
Albania	Culling	39%↓	Nocturnality
Croatia	Culling	43%↓	Nocturnality
Bosnia-Herzegovina	Hunting	51%↓	Nocturnality
Serbia	Hunting	44%↓	Nocturnality
Bulgaria	Hunting	25%↓	Nocturnality
Romania	Hunting	46%↓	Nocturnality
Hungary	—	28%↓	—
Slovak republic	Hunting	46%↓	Nocturnality
Austria	Region-dependent	41%↑	Nocturnality
**Czech Republic**	No	26%↓	**↑diurnality**
Macedonia	Hunting	42%↓	Nocturnality
Kosovo	—	—	—
Montenegro	—	33%↓	—
**Poland**	No	40%↓	**↑diurnality**

## References

[CIT0001] Bischof R. , Hameed S., Ali H., Kabir M., Younas M., Shah K.A., Din J.A., Nawaz M.A. 2014. Using time-to-event analysis to complement hierarchical methods when assessing determinants of photographic detectability during camera trapping. Methods in Ecology and Evolution 5:44–53.

[CIT0002] Blanco J.C. , Palomero G., Ballesteros F., López-Bao J.V. 2021. Habituation, food-conditioning and attacks on humans. In: Palomero G., Ballesteros F., Blanco J.C., López-Bao J.V., editors. Cantabrian bears. Demography, coexistence and conservation challenges. Brown Bear Foundation. Lynx Edicions, Barcelona; p. 65–89.

[CIT0003] Blumstein D.T. 2006. Developing an evolutionary ecology of fear: how life history and natural history traits affect disturbance tolerance in birds. Animal Behaviour 71:389–399.

[CIT0004] Braczhowski A. , O’Bryan C.J., Stringer M.J., Watson J.E., PossinghamH P., Hawthorne L.B. 2018. Leopards provide public health benefits in Mumbai, India. Frontiers in Ecology and the Environment 16:176–182.

[CIT0005] Carter N.H. , Linnell J.D.C. 2016. Co-adaptation is key to coexisting with large carnivores. Trends in Ecology and Evolution 31:575–578. 27377600 10.1016/j.tree.2016.05.006

[CIT0006] Chapron G. , et al. 2014. Recovery of large carnivores in Europe’s modern human-dominated landscapes. Science 346:1517–1519. 25525247 10.1126/science.1257553

[CIT0007] Chapron G. , López-Bao J.V. 2016. Coexistence with large carnivores informed by community ecology. Trends in Ecology and Evolution 31:578–580. 27377602 10.1016/j.tree.2016.06.003

[CIT0008] Chavez A.S. , Gese E.M. 2006. Landscape use and movements of wolves in relation to livestock in a wildland–agriculture matrix. Journal of Wildlife Management 70:1079–1086.

[CIT0009] Cimatti M. , et al. 2021. Large carnivore expansion in Europe is associated with human population density and land cover changes. Diversity and Distributions 27:602–617.

[CIT0010] Ciucci P. , Boitani L., Francisci F., Andreoli G. 1997. Home range, activity and movements of a wolf pack in central Italy. Journal of Zoology 243:803–819.

[CIT0011] Dennehy E. , Llaneza L., López-Bao J.V. 2021. Contrasting wolf responses to different paved roads and traffic volume levels. Biodiversity and Conservation 30:3133–3150.

[CIT0012] Flannery T. 2018. Europe: a natural history. Atlantic Monthly Press, New York, USA.

[CIT0013] Gaynor K.M. , Hojnowski C.E., Carter N.H., Brashares J.S. 2018. The influence of human disturbance on wildlife nocturnality. Science 360:1232–1235. 29903973 10.1126/science.aar7121

[CIT0014] Haswell P.M. , Kusak J., Jones K.A., Hayward M.W. 2020. Fear of the dark? A mesopredator mitigates large carnivore risk through nocturnality, but humans moderate the interaction. Behavioual Ecology and Sociobiology 74:62.

[CIT0015] Karlsson J. , Eriksson M., Liberg O. 2007. At what distance do wolves move away from an approaching human? Canadian Journal of Zoology 85:1193–1197.

[CIT0016] Kusak J. , Majić Skrbinsěk A., Huber D. 2005. Home ranges, movements, and activity of wolves (*Canis lupus*) in the Dalmatian part of Dinarids, Croatia. European Journal of Wildlife Research 51:254–262.

[CIT0017] Lagos L. , Bárcena F. 2015. EU sanitary regulation on livestock disposal: implications for the diet of wolves. Environmental Management 56:890–902. 26105972 10.1007/s00267-015-0571-4

[CIT0018] Lázaro A. 2014. Ecología trófica del lobo (*Canis lupus*) en un ambiente humanizado y multipresa: variación geográfica. M.Sc. thesis, University of Cordoba, Spain [Spanish].

[CIT0019] Llaneza L. , García E.J., Palacios V., Sazatornil V., López-Bao J.V. 2016. Resting in risky environments: the importance of cover for wolves to cope with exposure risk in human-dominated landscapes. Biodiversity and Conservation 25:1515–1528.

[CIT0020] Llaneza L. , López-Bao J.V. 2015. Indirect effect of changes in environmental and agricultural policies on the diet of wolves. European Journal of Wildlife Research 61:895–902.

[CIT0021] Llaneza L. , López-Bao J.V., Sazatornil V. 2012. Insights into wolf presence in human-dominated landscapes: the relative role of food availability, humans and landscape attributes. Diversity and Distributions 18:459–469.

[CIT0022] Llinares A. , Martínez-Abraín A., Veiga J. 2019. High foraging efficiency of Eurasian otters in a shallow Iberian reservoir. Wildlife Biology 2019:1–6.

[CIT0023] Manenti R. , Mori E., Di Canio V., Mercurio S., Picone M., Caffi M., Brambilla M., Ficetola G.F., Rubolini D. 2020. The good, the bad and the ugly of COVID-19 lockdown effects of wildlife conservation: insights from the first European locked down country. Biological Conservation 249:108728. 32863391 10.1016/j.biocon.2020.108728 PMC7441970

[CIT0024] Martínez-Abraín A. , Ferrer X., Jiménez J., Fernández-Calvo I.C. 2021. The selection of anthropogenic habitat by wildlife as an ecological consequence of rural exodus: empirical examples from Spain. Animal Biodiversity and Conservation 44:195–203.

[CIT0025] Martínez-Abraín A. , Jiménez J., Jiménez I., Ferrer X., Llaneza L., Ferrer M., Palomero G., Ballesteros F., Galán P., Oro D. 2020. Ecological consequences of human depopulation of rural areas on wildlife: a unifying perspective. Biological Conservation 252:108860.

[CIT0026] Martínez-Abraín A. , Jiménez J., Oro D. 2019b. New policies for a new wildlife: a road map for the wildlife manager of the future. Biological Conservation 236:484–488.

[CIT0027] Martínez-Abraín A. , Jiménez J., Oro D. 2019a. Pax Romana: “refuge abandonment” and spread of fearless behaviour in a reconciling world. Animal Conservation 22:3–13.

[CIT0028] Martínez-Abraín A. , Oro D. 2018. Nocturnality decreases under low human disturbance conditions. Science (E-letter, 3 July 2018, https://science.sciencemag.org/content/360/6394/1232/tab-e-letters). Accessed 18 December 2022.

[CIT0029] Martínez-Abraín A. , Oro D., Conesa D., Jiménez J. 2008. Compromise between seabird enjoyement and disturbance: the role of observed and observers. Environmental Conservation 35:104–108.

[CIT0030] Mech L.D. 1992. Daytime activity of wolves during the winter in northeastern Minnesota. Journal of Mammalogy 73:570–571.

[CIT0031] Mech L.D. 1997. The arctic wolf: ten years with the pack. Swan Hill, Detroit, Michigan.

[CIT0032] Mech L.D. , Adams L.G., Meier T.J., Burch H.W., Dale B.W. 1998. The wolves of Denali. University of Minnesota, Minneapolis, Minnesota, USA.

[CIT0033] Moen G.K. , Støen O.-G., Sahlén V., Swenson J.E. 2012. Behaviour of solitary adult Scandinavian brown bears (*Ursus arctos*) when approached by humans on foot. PLoS One 7:e31699. 22363710 10.1371/journal.pone.0031699 PMC3282762

[CIT0034] Møller A.P. , Ibánez-Álamo J.D. 2012. Escape behaviour of birds provides evidence of predation being involved in urbanization. Animal Behaviour 84:341–348.

[CIT0035] Navarro L.M. , Pereira H.M. 2012. Rewilding abandoned landscapes in Europe. Ecosystems 15:900–912.

[CIT0036] Rico M. , Torrente J.P. 2000. Caza y rarificación del lobo en España: investigación histórica y conclusiones biológicas. Galemys 12:163–179.

[CIT0037] Rosenzweig M. 2003. Win-win ecology: how the Earth’s species can survive in the midst of human enterprise. Oxford University Press, Oxford, United Kingdom.

[CIT0038] Sikes R. , and the Animal Care and Use Committee of the American Society of Mammalogists. 2016. 2016 Guidelines of the American Society of Mammalogists for the use of wild mammals in research and education. Journal of Mammalogy 97:663–688. 29692469 10.1093/jmammal/gyw078 PMC5909806

[CIT0039] Silliman B. , Hughes B.B., Gaskins L.C., He Q., Stepp R. 2018. Are the ghosts of nature’s past haunting ecology today? Current Biology 28:PR532–PR537. 10.1016/j.cub.2018.04.002 29738721

[CIT0040] Sterba J. 2012. Nature wars: the incredible story of how wildlife comebacks turned backyards into battlegrounds. Broadway Books, New York, USA.

[CIT0041] Swenson J.E. 1999. Does hunting affect the behaviour of brown bears in Eurasia? Ursus 11:157–162.

[CIT0042] Theuerkauf J. 2009. What drives wolves: fear or hunger? Humans, diet, climate and wolf activity patterns. Ethology 115:649–657.

[CIT0043] Theuerkauf J. , Jędrzejewski W., Schmidt K., Okarma H., Ruczyyński I., Śniezko S., Gula R. 2003. Daily patterns and duration of wolf activity in the Białowieza forest, Poland. Journal of Mammalogy 84:243–253.

[CIT0044] Wam H.K. , Eldegard K., Hjeljord O. 2012. From overlooking to concealed: predator avoidance in an apex predator. European Journal of Wildlife Research 58:1001–1003.

[CIT0045] Zedrosser A. , Steyaert S.M.J.G., Gossow H., Swenson J.E. 2011. Brown bear conservation and the ghost of persecution past. Biological Conservation 144:2163–2170.

